# The Dynamic Evolution Model of the Chemical and Carbon Isotopic Composition of C_1–3_ during the Hydrocarbon Generation Process

**DOI:** 10.3390/molecules29020476

**Published:** 2024-01-18

**Authors:** Heng Zhao, Yanjie Li, Wenhui Liu, Guchun Zhang, Yanjun Wang

**Affiliations:** 1 Jiangsu Design Institute of Geology for Mineral Resources (The Testing Center of China National Administration of Coal Geology (CNACG)), Xuzhou 221006, China; lyjdwyyx@163.com (Y.L.); guchun_zhang@163.com (G.Z.); yanjun2019_wang@163.com (Y.W.); 2 Department of Geology, Northwest University, Xi’an 710069, China; whliu@nwu.edu.cn

**Keywords:** dynamic evolution model, yield, carbon isotope, hydrocarbon generation, C_1–3_, geochemistry

## Abstract

A new approach is presented in this paper for the dynamic modeling of the chemical and isotopic evolution of C_1–3_ during the hydrocarbon generation process. Based on systematic data obtained from published papers for the pyrolysis of various hydrocarbon sources (type I kerogen/source rock, type II kerogen/source rock, type III kerogen/source rock, crude oil, and asphalt, etc.), the empirical evolution framework of the chemical and isotopic composition of C_1–3_ during the hydrocarbon generation process was built. Although the empirical framework was built only by fitting a large amount of pyrolysis data, the chemical and isotopic composition of C_1–3_ derived from the pyrolysis experiments all follow evolution laws, convincing us that it is applicable to the thermal evolution process of various hydrocarbon sources. Based on the simplified formula of the isotopic composition of mixed natural gas at different maturities (*δ*^13^C_mixed_), *δ*^13^C_mixed_ = $\frac{X{\times n}_{iA}{\times \delta }^{13}C_{iA}+Y{\times n}_{iB}{\times \delta }^{13}C_{iB}}{X{\times n}_{iA}+Y{\times n}_{iB}}$, it can be derived that the cumulative isotopic composition of alkane generated in a certain maturity interval can be expressed by the integral of the product of the instantaneous isotopic composition and instantaneous yield at a certain maturity point, and then divided by the cumulative yield of alkane generated in the corresponding maturity interval. Thus, the cumulative isotopic composition (A(X)), cumulative yield (B(X)), instantaneous isotope (C(X)), and instantaneous yield (D(x)) in the dynamic model, comply with the following formula during the maturity interval of (X_0_~X). A(X) = $\frac{\int_{X_{0}}^{X}C\left(X\right)\times D\left(X\right)dx}{B(X)}$, where A(X) and B(X) can be obtained by the fitting of pyrolysis data, and D(x) can also be obtained from the derivation of B(X). The dynamic model was applied on the pyrolysis data of Pingliang Shale to illustrate the quantitative evolution of the cumulative yield, instantaneous yield, cumulative isotope, and instantaneous isotope of C_1–3_ with increasing maturity. The dynamic model can quantify the yield of methane, ethane, and propane, as well as *δ*^13^C_1_, *δ*^13^C_2_, and *δ*^13^C_3_, respectively, during the hydrocarbon generation process. This model is of great significance for evaluating the natural gas resources of hydrocarbon source rock of different maturities and for identifying the origin and evolutionary process of hydrocarbons by chemical and isotopic data. Moreover, this model provides an approach to study the dynamic evolution of the isotope series of C_1–3_ (including reversed isotopic series), which is promising for revealing the mechanism responsible for isotopic reversal when combined with post-generation studies.

## 1. Introduction

The chemical and isotopic composition of light hydrocarbons have long been used to study the origins, generation, accumulation, and degradation process of hydrocarbons in the past decades [1,2,3,4,5,6,7,8,9]; however, with the large-scale exploration, development, and study of the intensive isotopic geochemistry of natural gas resources in the high evolution strata of superimposed basins, it is increasingly difficult to clearly identify the origin and evolutionary process of hydrocarbons by the chemical and isotopic characteristics of alkanes. This is because there are many factors and geochemical processes responsible for the chemical and isotopic compositions of hydrocarbons in geological conditions, such as the inheritance of isotopic signatures from precursor organics, kinetic isotope fractionations, and equilibrium isotope fractionations, and the mixing of hydrocarbons from different origins and of different maturities. Combined, these factors can readily lead to the overlapping of the bulk isotopic signatures of hydrocarbons of different origins and history, obscuring their origins and evolutionary processes. The mechanism responsible for the evolution of the isotopic composition of alkanes, e.g., the cause of the reversed alkane *δ*^13^C values in many natural gas plays, is still controversial and remains an open question [10,11,12,13].

The problems in the application of traditional natural gas isotope geochemistry can be attributed to the following two aspects: (a) for the current isotope analysis technology of monomer hydrocarbon, the isotopic information of the internal functional groups of the monomer hydrocarbon disappears in the process of oxidizing the target component to CO_2_ or reducing it to H_2_ and, accordingly, the intra-molecular isotope distribution information which can disclose the formation and evolution process of natural gas is not fully obtained [7,14,15]; (b) the dynamic evolution laws of the isotope composition of monomer hydrocarbon and corresponding mechanism during the formation and evolution process of natural gas are still in dispute [6,12,16,17]. Efforts to explore the intra-molecular isotope distribution of hydrocarbons, including position-specific isotope and clumped isotope analyses, have been made in the past decades [7,14,18,19,20]. This isotopic information with higher dimensions is expected to provide unique constraints on their formation and migration-degradation processes; however, the theoretical studies and applications of intra-molecular isotope distribution are still in their infancy and cannot be widely used as yet. The traditional monomer hydrocarbon isotope is still the main research method of natural gas isotope geochemistry; accordingly, research on the dynamic evolution of the chemical and isotopic composition of alkanes still needs to be strengthened, especially regarding dynamic evolution during the hydrocarbon generation process.

Efforts to study the dynamic evolution of the chemical and isotopic composition of alkanes (especially C_1–3_) during the hydrocarbon generation process have been made in the past decades. Many empirical models have been put forward to study the compositional and isotopic variation of natural gas [1,21,22,23]; however, these empirical models were developed based on the statistical analysis of massive data, and the mechanisms responsible for the compositional and isotopic variation of alkanes were not fully understood. The Rayleigh model is the first semi-quantitative model to study the compositional and isotopic variation of alkanes [24,25,26], but the Rayleigh model cannot exactly study the compositional and isotopic variation of alkanes. The obvious defects of the Rayleigh model are as follows [3]: one is the assumption that methane and the higher hydrocarbons can be modeled using a single extent of reaction parameter; the other is the assumption that the fractionation factor of each first-order reaction is constant. The hydrocarbon generation kinetic model based on various pyrolysis experiments is widely applied to the quantitative study of the yield evolution of methane and total gaseous hydrocarbon (C_1_–C_5_),while the carbon isotope kinetic model based on various pyrolysis experiments is widely applied to the quantitative study of the isotopic evolution of methane during the hydrocarbon generation process [3,27,28,29,30,31,32]. The hydrocarbon generation kinetic model and carbon isotope kinetic model are only applicable to the first-order reaction in the hydrocarbon generation process. The yield of total gaseous hydrocarbon (C_1–5_) can also reach a plateau in hydrocarbon generation simulation experiments, so C_1–5_ can be considered as a whole to calculate its yield evolution using hydrocarbon generation simulation data. However, the yield of individual heavy hydrocarbon components initially increases and then decreases after reaching maximum yield in hydrocarbon generation simulation experiments, indicating that the heavy hydrocarbon components have undergone pyrolysis. In addition, the heavy hydrocarbon components may involve a Fischer–Tropsch reaction in the high evolution stage, so individual heavy hydrocarbon components cannot be regarded as a first-order reaction and are not suitable for the hydrocarbon generation kinetic model and carbon isotope kinetic model. Tang et al. (2000) predicted the evolution of the cumulative yield of methane, instantaneous yield of methane, instantaneous *δ*^13^C_1_, and cumulative *δ*^13^C_1_ by the hydrocarbon generation kinetic model and carbon isotope kinetic model, based on the pyrolysis of n-octadecane [3]. Shuai et al. (2005, 2006) carried out the hydrocarbon generation kinetic and carbon isotope kinetic studies of ethane by dividing the evolution process of ethane into two stages [33,34]: the generation-dominated stage and the cracking-dominated stage. However, each stage simultaneously involves the generation and cracking of ethane, and there may be ethane generated by the Fischer–Tropsch reaction in the cracking-dominated stage. To sum up, there is no valid model to quantitatively study the chemical and isotopic variation of C_1–3_ during the hydrocarbon generation process to date, making it very difficult to identify the origin, source, and resources of natural gas by chemical and isotopic data.

To address this limitation, a quantitative model was built to illustrate the chemical and isotopic variation of C_1–3_ during the process of hydrocarbon generation.

The objective of this work is multifold:

(1) Summarize the universal evolution laws of the chemical and isotopic composition of C_1–3_ in the hydrocarbon generation process on the basis of fitting systematic data obtained from published papers on the pyrolysis of various hydrocarbon sources;
(2) Introduce the theoretical approach and detailed steps to build the dynamic model of the chemical and isotopic evolution of C_1–3_ during the hydrocarbon generation process;
(3) Illustrate the application of the dynamic model to the pyrolysis experiment of the Ordovician Pingliang Shale from the Ordos Basin, China, to study the chemical and isotopic evolution of C_1–3_ during the hydrocarbon generation process.

## 2. Results and Discussion

The quantitative model was applied on the anhydrous-confined pyrolysis data of the kerogen extracted from the Ordovician Pingliang Shale in the Ordos Basin, China [31], to study the chemical and isotopic evolution of C_1_–C_3_ during the hydrocarbon generation process. Detailed information regarding the Ordovician Pingliang Shale is as follows: marine shale, type II kerogen, TOC 18.1%, vitrinite reflectance (R_O_) 0.7%. The *δ*^13^C of the isolated kerogen is 30.1‰. The detailed procedures of the anhydrous-confined pyrolysis experiment are as follows: the kerogen samples were introduced into gold tubes and were sealed under argon; the tubes containing fresh kerogen were loaded into a stainless vessel; pyrolysis was performed under a constant pressure of 50 MPa, with pyrolysis temperatures ranging from 250 to 600 °C at heating rates of 2 °C/h [35]. The simulation temperature was converted to “Easy Ro” to represent the evolutionary maturity of the Pingliang Shale [36].

**Figure 1:**
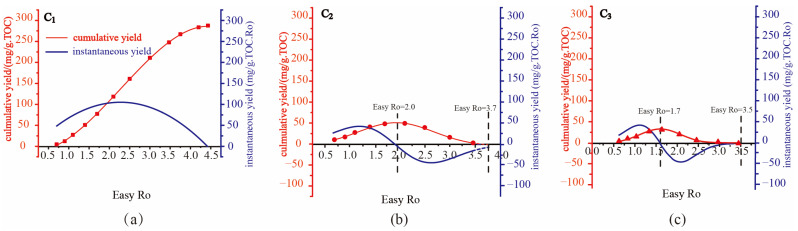
The instantaneous and cumulative yield of C_1–3_ from the pyrolysis of the Pingliang Shale at different maturities, squares in subfigure (**a**) represents cumulative and instantaneous yield of methane, circulars in subfigure (**b**) represents cumulative and instantaneous yield of ethane, and triangles in subfigure (**c**) represents cumulative and instantaneous yield of propane.

**Figure 2:**
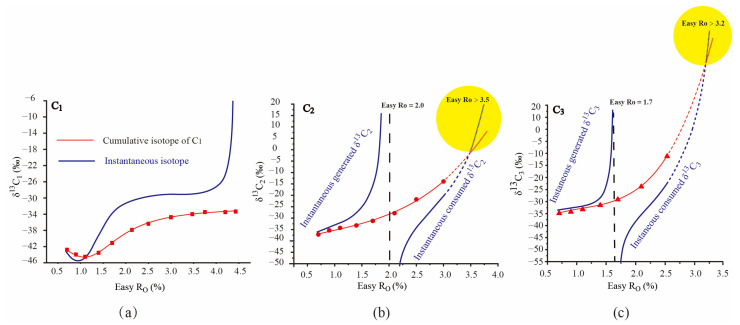
The instantaneous and cumulative isotope of C_1–3_ from the pyrolysis of the Pingliang Shale at different maturities, squares in subfigure (**a**) represents cumulative and instantaneous isotope of methane, circulars in subfigure (**b**) represents cumulative and instantaneous isotope of ethane, and triangles in subfigure (**c**) represents cumulative and instantaneous isotope of propane.

The cumulative isotope function (A(X)) and cumulative yield function (B(X)) of kerogen from the Pingliang Shale are obtained by the data fitting of confined pyrolysis experiments (Figure 1 and Figure 2). The instantaneous yield function (D(x)) can be obtained by taking the derivative of the cumulative yield function (B(X)); the instantaneous isotope function (C(x)) can be calculated by Formula (6) (Figure 1 and Figure 2). The unit of the instantaneous yield function [mg/(g·TOC·Ro)] is different from the unit of the cumulative yield function [mg/(g·TOC)], so the charts for the instantaneous and cumulative yields were plotted using the dual-axis method, and there is no direct comparability between the value of the instantaneous and cumulative yields.

### 2.1. Dynamic Chemical Evolution of C_1–3_ during Hydrocarbon Generation Process

#### 2.1.1. Dynamic Evolution of C_1_ Yield during Hydrocarbon Generation Process

The cumulative yield of C_1_ originating from the pyrolysis of the Pingliang Shale increases continuously with rising maturity and reaches a plateau at the end of the methane generation process, which is consistent with the result that the instantaneous yield of C_1_ always stays positive during the methane generation process. The cumulative yield of C_1_ increases quite mildly at two ends of the curve and increases quite sharply in the middle of the curve, which is consistent with the result that the instantaneous yield of C_1_ initially increases and then decreases with raising maturity, reaching maximum instantaneous yield at a certain intermediate maturity (Easy Ro approx. 2.4%).

Methane is widespread in various stages of the hydrocarbon generation evolution of the Pingliang Shale: in the immature stage, low-mature stage, mature stage, high-mature stage, and over-mature stage. At the immature stage, low mature stage and mature stage, liquid hydrocarbons dominate the hydrocarbon generation product of the sapropelic Pingliang Shale. The gaseous hydrocarbons originating mainly from the degradation of the aliphatic side chain only account for a very low proportion of the total hydrocarbons, so the instantaneous and cumulative yield of C_1_ is quite low at the beginning of the hydrocarbon generation process. At the high-mature stage, liquid hydrocarbons start to be extensively cracked, and crude oil cracking gas becomes the dominant origin of gaseous hydrocarbon, while the proportion of kerogen cracking gas decreases quickly. Since the hydrocarbon-generating intensity of crude oil cracking is 3–4 times that of kerogen cracking [37,38], the instantaneous yield of C_1_ increases sharply and reaches maximum at Easy Ro approx. 2.4% in this period. At the over-mature stage, liquid hydrocarbons are mostly cracked and the gas generation potential of kerogen is also nearly exhausted, so the instantaneous yield of C_1_ decreases sharply at the end of the over-mature stage [38]. At Easy Ro approx. 4.5%, the cumulative yield of C_1_ reaches a plateau as the instantaneous yield of C_1_ approaches zero, implying that the hydrocarbon generation potential of the Pingliang Shale is already exhausted at Easy Ro approx. 4.5%. However, the instantaneous yield of C_1_ has never become negative during the whole hydrocarbon generation evolution stage, indicating that there is no cracking of methane. Previous research on the calculation of the reaction activation energy and pyrolysis experiments of methane have also proven that methane is so thermodynamically stable that it can hardly be pyrolyzed under geological conditions and hydrocarbon generation simulation experiments [38,39,40]. Previous calculations have shown that the activation energy required for methane cracking is 441 kJ/mol, higher than that of ethane and propane [39,40]. Moreover, it has been verified by extensive pyrolysis experiments on methane that the threshold temperature of methane pyrolysis is 1100 °C at atmospheric pressure (equivalent Easy Ro approx. 5.0%) [38]. The maximum temperature of the pyrolysis experiment on the Pingliang Shale is 600 °C, far lower than the threshold temperature of methane pyrolysis. It follows that the instantaneous yield of C_1_ is promising for predicting the maturity deadline of hydrocarbon generation, which has practical significance for defining the upper limit of natural gas exploration.

#### 2.1.2. Dynamic Evolution of C_2–3_ Yield during Hydrocarbon Generation Process

The variation trends of the cumulative yields C_2_ and C_3_ are identical: the cumulative yields initially increase and then decrease with increasing thermal maturity, reaching maximum yields at a certain intermediate maturity. C_2_ and C_3_ reach maximum cumulative yield at Easy Ro approx. 2.0% and 1.7%, respectively (Figure 1). At the same time, the instantaneous yield of C_2_ and C_3_ shifts from positive to negative values at Easy Ro 2.0% and 1.7%, respectively, which indicates the cracking threshold maturity of C_2_ and C_3_. Actually, both the generation and cracking of C_2_ and C_3_ are present before and after the threshold maturity; C_2_ and C_3_ shift from the generation-dominated stage to the cracking-dominated stage at Easy Ro 2.0% and 1.7%, respectively. For simplicity, it is assumed in this study that there is only generation of C_2_ and C_3_ before the threshold maturity (Easy Ro 2.0% and 1.7%, respectively), and there is only cracking of C_2_ and C_3_ after the threshold maturity.

Propane starts cracking at Easy Ro 1.7% and ends cracking at Easy Ro 3.5%, while ethane starts cracking at Easy Ro 2.0% and ends cracking at Easy Ro 3.7%. Previous studies have shown that the activation energy required for ethane cracking is higher than that of propane [38]; therefore, the later cracking of ethane can probably be attributed to its higher molecular stability. The variation trends of the instantaneous yields of C_2_ and C_3_ are also identical. During the generation stage (instantaneous yield > 0), the instantaneous yield initially increases and then decreases with raising maturity, reaching maximum instantaneous yield at a certain intermediate maturity. The increase of the instantaneous yield of C_2_ and C_3_ at the early-generation stage can mainly be attributed to the result that C_2_ and C_3_ originating from kerogen cracking and crude oil cracking begins to be extensively generated at the mature and high-mature stages. The decrease of the instantaneous yield of C_2_ and C_3_ at the late-generation stage can mainly be attributed to the result that kerogen cracking and crude oil cracking are more inclined to generate C_1_ rather than C_2_ and C_3_ at the high-mature stage. During the consumption stage (instantaneous yield < 0), the instantaneous consumption rate initially increases and then decreases with raising maturity, reaching maximum instantaneous consumption rate at a certain intermediate maturity. The increase of the instantaneous consumption rate at the early-consumption stage is owing to the initiation of the extensive cracking of C_2_ and C_3_ at the high-mature stage and over-mature stage. The decrease of the instantaneous consumption rate at the late-consumption stage is owing to the result that the cumulative yields of C_2_ and C_3_ are mostly cracked and cracking potential of C_2_ and C_3_ is nearly exhausted.

### 2.2. Dynamic Isotopic Evolution of *δ*^13^C_1–3_ during Hydrocarbon Generation Process

#### 2.2.1. Dynamic Isotopic Evolution of *δ*^13^C_1_ during Hydrocarbon Generation Process

**Figure 3:**
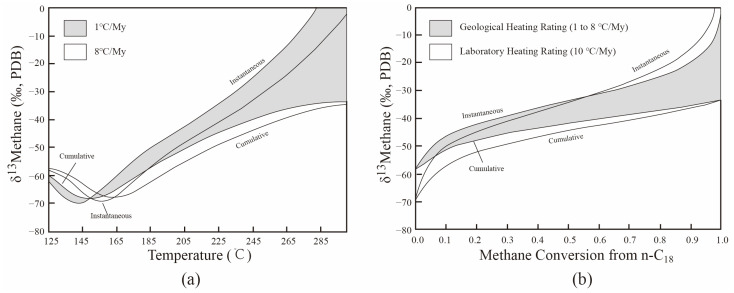
The calculated evolution trend of instantaneous and cumulative *δ*^13^C_1_ [3]. subfigure (**a**) represents predicted isotopic composition versus temperature for instantaneous and cumulative methane generated from pyrolysis of n-octadecane; subfigure (**b**) represents Calculated isotopic composition versus gas yield for representative geological and laboratory heating rates.

**Figure 4:**
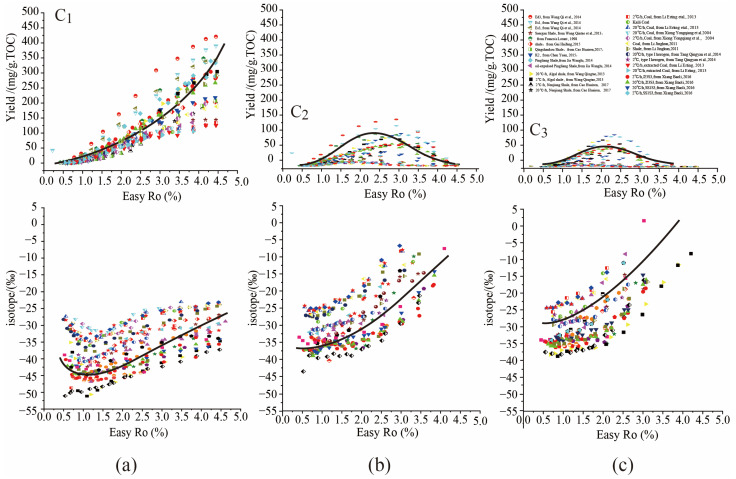
The universal evolution laws summarized from massive hydrocarbon generation simulation experiments [26,28,30,31,32,33,46,47,48,49,50,51,52,53,54,55,56,57,58]. Subfigure (**a**) represents cumulative yield and isotopic composition of methane generated from pyrolysis experiments, subfigure (**b**) represents cumulative yield and isotopic composition of ethane generated from pyrolysis experiments, subfigure (**c**) represents cumulative yield and isotopic composition of propane generated from pyrolysis experiments.

The cumulative and instantaneous *δ*^13^C_1_ from the pyrolysis of the Pingliang Shale all present identical variation patterns: they shift negatively at the early hydrocarbon generation stage, and then shift positively until the end of methane generation. The variation trend of cumulative *δ*^13^C_1_ depends on the relative isotopic composition values between cumulative *δ*^13^C_1_ and instantaneous *δ*^13^C_1_: when instantaneous *δ*^13^C_1_ is lighter than cumulative *δ*^13^C_1_ (Easy Ro < 1.1%), the cumulative *δ*^13^C_1_ shifts negatively; when instantaneous *δ*^13^C_1_ is heavier than cumulative *δ*^13^C_1_ (Easy Ro > 1.1%), the cumulative *δ*^13^C_1_ shifts positively. The rollover of cumulative and instantaneous *δ*^13^C_1_ have already be verified by the study of Tang et al. (2000), which used the carbon isotope kinetic model (Figure 3a) [3]. Moreover, the rollover of cumulative *δ*^13^C_1_ from the pyrolysis of the Pingliang Shale was also observed in many pyrolysis experiments [26,31,41,42,43,44,45] (Figure 4).

The mechanism responsible for the isotope rollover of *δ*^13^C_1_ involves the presence of two or more precursors for methane generation with different isotopic compositions, and the mixing of methane originating from different precursors, which leads to the isotope rollover of C_1_ [3]. Heteroatoms such as O and S are widely present in organic matter, and alkyl carbons connected to these heteroatoms are isotopically heavier. Due to the instability of the C–O or C–S bonds, these isotopically heavier alkyl groups were preferentially decomposed to form isotopically heavier methane. When the alkyl carbons connected to heteroatoms were almost cracked, the isotopically lighter methane originating from the cracking of more tightly-bound C–C bonds was extensively generated. The initial trend of decreasing *δ*^13^C_1_ values can be explained by the mixing of isotopically lighter methane. For alkyl groups connected with the C–C bond, ^12^C-enriched alkyl groups were preferentially decomposed to form isotopically lighter methane. With increasing evolution degree, ^13^C-enriched alkyl groups were gradually decomposed to form isotopically heavier methane, resulting in the increase of *δ*^13^C_1_. However, the rollover of *δ*^13^C_1_ was also observed in many pyrolysis experiments on pure n-alkanes, which are free of heteroatoms, indicating that this theory cannot cover all of the *δ*^13^C_1_ rollover occurrences. There are two sets of methane precursors with significantly different activation energies in the hydrocarbon source, and the precursor with the lower activation energy typically has a higher isotope fractionation factor and tends to generate isotopically lighter methane, while the precursor with the higher activation energy typically has a lower isotope fractionation factor and tends to generate isotopically heavier methane [3]. Since the precursor with the lower activation energy was preferentially decomposed to form isotopically heavier methane, the variation trends of *δ*^13^C_1_ initially decrease with the mixing of isotopically lighter methane originating from the decomposition of the precursor with the higher activation. When the precursor with the lower activation was almost decomposed, the precursor with the higher activation was gradually decomposed to form isotopically heavier methane, leading to the increase of *δ*^13^C_1_ [6].

A sudden and sharp increase of instantaneousδ^13^C_1_ was observed at the end of the methane generation stage, but the cumulative *δ*^13^C_1_ shows an only very gentle increase at this stage. The sharply increasing instantaneous *δ*^13^C_1_ was also observed when the methane conversion rate approached 1.0 in the study of Tang et al. (2000) (Figure 3b) [3]. In addition, Liu and Xu (1999) also documented the sudden and sharp increase of *δ*^13^C_1_ in 106 coal-type gas samples from 10 basins in China at the high-mature and over-mature stages, and a two-stage model of carbon isotopic fractionation was built to explain the fractionation mechanism of *δ*^13^C_1_ [23]. As mentioned in Section 2.1.1, methane is mainly generated from the cracking of kerogen, and the instantaneous yield is very limited at the end of the methane generation stage; moreover, there is no cracking of methane in this period. Accordingly, the sudden and sharp increase of instantaneous *δ*^13^C_1_ may be attributed to the sudden transformation of the precursor with the higher activation energy and lower isotope fractionation factor. Since previous normal methane precursors have been mostly cracked at the end of the methane generation stage, the continuously advancing evolutionary process may suddenly result in the cracking of the unconventional precursor with the higher activation energy, leading to the sudden and sharp increase of instantaneous *δ*^13^C_1_. As the instantaneous C_1_ yield is very limited in this period, the sharp increase of instantaneous *δ*^13^C_1_ did not lead to the obvious increase of cumulative *δ*^13^C_1_.

#### 2.2.2. Dynamic Isotopic Evolution of *δ*^13^C_2–3_ during Hydrocarbon Generation Process

The cumulative *δ*^13^C_2_/δ^13^C_3_ and instantaneous *δ*^13^C_2_/δ^13^C_3_ from the pyrolysis of the Pingliang Shale all present an identical variation pattern: a cumulative *δ*^13^C_2_/δ^13^C_3_ increase continuously with increasing maturity. The instantaneous *δ*^13^C_2_/δ^13^C_3_ present a two-stage pattern (Figure 2). The instantaneous *δ*^13^C_2_/δ^13^C_3_ in the generation stage (instantaneous yield > 0) mainly represents the isotope of instantaneously generated C_2_/C_3_, while the instantaneous *δ*^13^C_2_/δ^13^C_3_ in the consumption stage (instantaneous yield < 0) mainly represents the isotope of instantaneously cracked C_2_/C_3_. The instantaneously generated *δ*^13^C_2_/δ^13^C_3_ increase with raising maturity until the end of C_2_/C_3_ generation, which is the result of the preferential generation of ^12^C-enriched C_2_/C_3_. The instantaneously consumed *δ*^13^C_2_/δ^13^C_3_ increase with raising maturity until the exhaustion of C_2_/C_3_, which can all be attributed to the preferential decomposition of ^12^C-enriched C_2_/C_3_. This is similar to the variation trend of instantaneous *δ*^13^C_1_ at the end of the methane generation stage. The instantaneously generated *δ*^13^C_2_/δ^13^C_3_ also present a sudden and sharp increase at the end of the C_2_/C_3_ generation stage, which may also be attributed to the cracking of the unconventional precursor with the higher activation energy.

The variation trend of cumulativeδ^13^C_2_/δ^13^C_3_ was controlled by the relative isotopic composition values between cumulativeδ^13^C_2_/δ^13^C_3_ and instantaneously generated *δ*^13^C_2_/δ^13^C_3_ in the generation stage, as well as the relative isotopic composition values between cumulative *δ*^13^C_2_/δ^13^C_3_ and instantaneously consumed *δ*^13^C_2_/δ^13^C_3_ in the consumption stage. In the generation stage, the instantaneously generated *δ*^13^C_2_/δ^13^C_3_ is heavier than the corresponding cumulative *δ*^13^C_2_/δ^13^C_3_; accordingly, the cumulative *δ*^13^C_2_/δ^13^C_3_ increase continuously with raising maturity. In the consumption stage, the instantaneously consumed *δ*^13^C_2_/δ^13^C_3_ is lighter than the corresponding cumulative *δ*^13^C_2_/δ^13^C_3_; accordingly, the cumulative *δ*^13^C_2_/δ^13^C_3_ still increase continuously with raising maturity. As commonly acknowledged, the bond energy of ^12^C-^12^C is lighter than that of ^12^C-^13^C and ^12^C-^13^C, so ^12^C-enriched C_2_/C_3_ should preferentially be decomposed during the consumption stage. As a result, the *δ*^13^C of residual *δ*^13^C_2_/δ^13^C_3_ (cumulative *δ*^13^C_2_/δ^13^C_3_) should be heavier than instantaneously consumed *δ*^13^C_2_/δ^13^C_3_ during the consumption stage. This is the case observed in most of the consumption stages for C_2_/C_3_ but, at the end of the consumption stage, the instantaneously consumed *δ*^13^C_2_/δ^13^C_3_ reversed to be more positive than cumulative *δ*^13^C_2_/δ^13^C_3_ (yellow labelled area in Figure 2b,c), which is unreasonable according to the bond energy theory. The reversal of instantaneously consumed *δ*^13^C_2_/δ^13^C_3_ and cumulative *δ*^13^C_2_/δ^13^C_3_ happened when the cumulative C_2_ and C_3_ have almost been decomposed (Easy Ro > 3.5% and 3.2%, respectively). As shown in Figure 2, the reversal of instantaneously consumed ^13^C_2_/δ^13^C_3_ and cumulative *δ*^13^C_2_/δ^13^C_3_ can mainly be ascribed to the sudden and sharp increase of instantaneously consumed ^13^C_2_/δ^13^C_3_, which is similar to the sudden and sharp increase of instantaneously generated *δ*^13^C_1_. Accordingly, it can be speculated that the sudden and sharp increase of instantaneously consumed ^13^C_2_/δ^13^C_3_ may also be attributed to the sudden transformation of an unconventional precursor, e.g., propane analogues with unconventionally heavier intra-molecular isotope distribution and higher activation energy, when common precursors have been mostly cracked.

## 3. Theoretical Approach

### 3.1. The Universal Chemical and Isotopic Evolution Laws of C_1–3_ in Hydrocarbon Generation Simulation Experiments

The universal evolution laws of hydrocarbon composition and isotopes were summarized by the data fitting of massive hydrocarbon generation simulation experiments involving various hydrocarbon sources, obtained from the available literature, including type I kerogen/source rock, type II kerogen/source rock, type III kerogen/source rock, crude oil, and asphalt, etc. [26,28,30,31,32,33,46,47,48,49,50,51,52,53,54,55,56,57,58] (Figure 4). For the evolution laws of C_1–3_ yields, C_1_ yields increase continuously with rising maturity, while C_2_ and C_3_ yields initially increase and then decrease with increasing thermal maturity, reaching maximum yields at a certain intermediate maturity. As regards the evolution laws of *δ*^13^C_1–3_, *δ*^13^C_1_ presents obvious isotope rollover, initially shifting negatively and then shifting positively with increasing maturity; *δ*^13^C_2_ and *δ*^13^C_3_ in certain simulation experiments also present isotope rollover with increasing maturity, but the variation amplitude is much lower than that of *δ*^13^C_1_. Overall, *δ*^13^C_2_ and *δ*^13^C_3_ generally shift positively with increasing maturity (Figure 4). 

Since the samples adopted in the hydrocarbon generation simulation experiments are of different organic matter type and different maturities, the yields and isotope compositions of C_1–3_ may vary greatly from each other; however, the yields and isotope compositions of C_1–3_ present universal variation trends. Although these universal laws are empirical models summarized from massive data statistics (only the variation of C_1_ yields and *δ*^13^C_1_ values are verified by hydrocarbon generation kinetics and isotope kinetics), the massive hydrocarbon generation simulation data of various hydrocarbon sources all comply with these universal laws, convincing us that these universal laws are applicable to the hydrocarbon generation process of various organic matter. 

### 3.2. Calculation of Isotopic Composition of Mixed Natural Gas from Different Maturities (δ^13^C_mixed_)

The carbon isotope of mixed natural gas at different maturities can be formulated by Equations (1) and (2), according to the definition of the natural gas isotope.

(1) $$\delta^{13}C=[\frac{R_{sample}}{R_{standard}}-1]\times 1000(‰)$$

where R denotes the isotope ratio of ^13^C/^12^C of the PDB standard.

(2) $$\delta^{13}C_{\mathrm{mixed}}=\frac{X{\times n}_{iA}*\Delta_{iA}\times (1+\Delta_{iB})+Y{\times n}_{iB}\times \Delta_{iB}\times (1+\Delta_{iA})}{X{\times n}_{iA}\times \left(1+\Delta_{iB}\right)+Y{\times n}_{iB}\times (1+\Delta_{iA})}$$

A and B represent different natural gas end members; X and Y represent the proportion of A and B in mixed natural gas, respectively; n_i_ represents the concentration of alkane component i in natural gas; *δ*^13^C_i_ represents the carbon isotope of component i; Δ = *δ*^13^C/1000 + 1; R represents the ^13^C/^12^C ratio of the PDB standard. However, too many parameters in Equation (2) make it too complex to calculate *δ*^13^C_mixed_.

(3) $$\delta^{13}C_{\mathrm{mixed}}=\frac{X{\times n}_{iA}{\times \delta }^{13}C_{iA}+Y{\times n}_{iB}{\times \delta }^{13}C_{iB}}{X{\times n}_{iA}+Y{\times n}_{iB}}$$

Xia et al. (1998) put forward a simplified formula to study the influence of mixture on natural gas isotopes (Equation (3)) [59]. Although the simplified formula has been widely applied to the isotopic study of natural gas, some researchers are still skeptical of this formula and insist that it is only applicable to limited proportions of end members and may lead to obvious mistakes beyond limited proportions. The comparative study of the definition formula of mixed natural gas (Equation (2)) and the simplified formula (Equation (3)) were conducted under different proportions (A:B = 1:9999~9999:1) to verify the validity of the simplified formula. To strengthen reliability and persuasion, the scope between *δ*^13^C_iA_ and *δ*^13^C_iB_ should cover the variation range of *δ*^13^C_i_ in geological and experimental conditions. In addition, the difference between *δ*^13^C_iA_ and *δ*^13^C_iB_ should be big enough. Accordingly, we assign *δ*^13^C_iA_ and *δ*^13^C_iB_ to be −10‰ and −50‰, respectively. As shown in Table 1, the *δ*^13^C_mixed_ values calculated according to the simplified formula are very close to those calculated according to the definition formula under obviously different proportions, with only a minor difference on the fifth significant digit. To date, the precision of one digit after the decimal point for *δ*^13^C is sufficient to meet the requirement of the isotope study of natural gas. So, the simplified formula of *δ*^13^C_mixed_ can be widely applied to the isotopic study of natural gas under diverse proportions of different end members.

### 3.3. Dynamic Modeling of Chemical and Isotopic Variation of C_1–3_ during Hydrocarbon Generation Process

#### 3.3.1. Nomenclature

(1) Cumulative yield: Total yield of certain alkane gas in certain maturity interval or geological period;
(2) Instantaneous yield: Yield of certain alkane gas at certain single maturity point or single point of geological time;
(3) Cumulative isotope: Isotopic composition of alkane generated in certain maturity interval or geological period;
(4) Instantaneous isotope: Isotopic composition of alkane generated at certain single maturity point or single point of geological time.

Supposing the following: the cumulative isotope function of X (maturity) is A(X); the cumulative yield function of X (maturity) is B(X); the instantaneous isotope function of X (maturity) is C(X); the instantaneous yield function of X (maturity) is D(x).

#### 3.3.2. Calculations

According to the simplified formula of *δ*^13^C_mixed_ (Formula (3)), we can infer that A(X), B(X), C(X), and D(x) comply with Formula (4) in the maturity interval of (X_0_~X), based on the theory of integration and differentiation:

(4) $$A(X)=\frac{\int_{X_{0}}^{X}C\left(X\right)\times D\left(X\right)dx}{B(X)}$$

From Equation (4), we have

(5) $$A(X)\times B(X)=\int_{X_{0}}^{X}C\left(X\right)\times D\left(X\right)dx$$

Derivatives were taken on both sides of Formula (5), we have

(6) $$C(X)=\frac{d[A(X)\times B(X)]}{D(X)}$$

The cumulative isotope function (A(X)) and cumulative yield function (B(X)) can be obtained by the data fitting of the yield and isotope data from pyrolysis experiments using Origin software (https://www.originsoftware.net/). The conventional viewpoints insist that the yields and isotopes of alkanes collected at certain temperatures/maturity points in open pyrolysis experiments can represent the instantaneous yield and instantaneous isotope, respectively. However, the alkanes collected at certain temperatures/maturities in open pyrolysis experiments are actually found to be the cumulative pyrolysis product during the maturity intervals of two sampling points (except when using the on-line pyrolysis-GC-IRMS method). Although alkanes collected at certain maturities in open pyrolysis experiments can reflect the variation trends of instantaneous yields and instantaneous isotopes when the sampling interval is very small, they cannot accurately represent the instantaneous yield and instantaneous isotope. In this study, the instantaneous yield function (D(x)) can be obtained by taking the derivative of the cumulative yield function (B(X)), and the instantaneous isotope function (C(x)) can be calculated by Formula (6). The calculation and plotting of Formula (6) were conducted using Maple software (https://www.maplesoft.com/products/Maple/).

It has been documented that hydrocarbon generation history (including heating rate (subsidence rate), geothermal gradient, and primary/secondary hydrocarbon generation) also imposes significant influence on the compositional and isotopic characteristics of hydrocarbon [3,60,61]; however, for simplicity, it is assumed in this study that hydrocarbon generation history does not influence the instantaneous yield and isotope characteristics of hydrocarbon at each maturity. The above-mentioned cumulative yield function (B(X)) and cumulative isotope function (A(X)) are obtained based on the fact that all pyrolysis products are preserved in a confined pyrolysis system. For natural gas systems which experienced hydrocarbon expulsion, the calculation of the cumulative isotope (A(X)) must take into consideration the expulsion ratio on the basis of Formula (4), which is discussed elsewhere (Zhao et al., in prep).

## 4. Conclusions

Based on systematic data obtained from published papers on the pyrolysis of various hydrocarbon sources, the empirical evolution framework of the chemical and isotopic composition of C_1–3_ generated from various organic matter was built, which laid an overall foundation for the dynamic evolution model of the chemical and isotopic composition of C_1–3_ in this study.

Based on the calculation of the isotopic composition of mixed natural gas at different maturities (δ^13^C_mixed_), the quantitative relationship between the cumulative isotopic composition (A(X)), cumulative yield (B(X)), instantaneous isotope (C(X)), and instantaneous yield (D(x)), were accomplished according to integration theory, which is the core of the dynamic model. Moreover, the dynamic model was applied to the pyrolysis data of the Pingliang Shale, and the cumulative yield, instantaneous yield, cumulative isotope, and instantaneous isotope were illustrated to study the chemical and isotopic evolution of C_1–3_ during the hydrocarbon generation process

The geological significance of the dynamic model are as follows:

(1) Quantification of the yield and proportion of methane, ethane, and propane during the hydrocarbon generation process by the parameters of the cumulative yield of C_1–3_ and instantaneous yield of C_1–3_, providing a basis for the evaluation of natural gas resources from hydrocarbon source rock of different maturities, especially the evaluation of high-mature source rock.
(2) Clarification of the cracking maturity of ethane and propane, and the proposal that there is no cracking of methane below the Easy Ro of 4.5%, which may be helpful for the natural gas exploration of deep formations.
(3) Quantification of the evolution of *δ*^13^C_1_, *δ*^13^C_2_, and *δ*^13^C_3_ during the hydrocarbon generation process by the parameters of cumulative ^13^C_1–3_ and instantaneous ^3^C_1–3_, improving the accuracy of identifying the origin and evolutionary process of hydrocarbons by chemical and isotopic data, making it possible to study the dynamic evolution of the isotope series of C_1–3_ (including reversed alkane series).

The empirical evolution framework of the chemical and isotopic composition of C_1–3_, which was built on the basis of the data fitting of systematic pyrolysis experiments on different hydrocarbon sources, laid a factual basis for the dynamic model in this study. However, the theoretical basis is still weak; accordingly, the mechanism responsible for the evolution trends of the cumulative yield, instantaneous yield, cumulative isotope, and instantaneous isotope, still needs to be systematically and deeply studied.

## Figures and Tables

**Table 1:** The *δ*^13^C_mixed_ value of mixed natural gas with different proportions of end members A and B.

Proportion of End Member A (%)	Proportion of End Member B (%)	*δ*^13^C_mixed_ according to Definition Formula (‰)	*δ*^13^C_mixed_ according to Simplified Formula (‰)
0.01	99.99	−49.996	−49.996
0.1	99.9	−49.960	−49.960
1	99	−49.600	−49.600
5	95	−48.001	−48.000
10	90	−46.002	−46.000
20	80	−42.003	−42.000
30	70	−38.004	−38.000
40	60	−34.004	−34.000
50	50	−30.004	−30.000
60	40	−26.004	−26.000
70	30	−22.004	−22.000
80	20	−18.003	−18.000
90	10	−14.002	−14.000
95	5	−12.001	−12.000
99	1	−10.400	−10.400
99.9	0.1	−10.040	−10.040
99.99	0.01	−10.004	−10.004

## Data Availability

The data presented in this study are available on request from the corresponding author. The data are not publicly available due to confidentiality.

## References

[1] Schoell M. (1980). The hydrogen and carbon isotopic composition of methane from natural gases of various origins. Geochim. Cosmochim. Acta.

[2] Rooney M.A., Claypool G.E., Chung H.M. (1995). Modeling thermogenic gas generation using carbon isotope ratios of natural gas hydrocarbons. Chem. Geol..

[3] Tang Y., Perry J., Jenden P., Schoell M. (2000). Mathematical modeling of stable carbon isotope ratios in natural gases. Geochim. Cosmochim. Acta.

[4] Galimov E. (2006). Isotope organic geochemistry. Org. Geochem..

[5] Liu Q., Worden R., Jin Z., Liu W., Li J., Gao B., Zhang D., Hu A., Yang C. (2012). TSR versus non-TSR processes and their impact on gas geochemistry and carbon stable isotopes in Carboniferous, Permian and Lower Triassic marine carbonate gas reservoirs in the Eastern Sichuan Basin, China. Geochim. Cosmochim. Acta.

[6] Zhao H. (2019). The Isotopic Evolution of Natural Gas During Accumulation Process—A Model of Differential Accumulation and Loss of Hydrocarbon for Isotope Reversal.

[7] Zhao H., Liu C., Larson T.E., McGovern G.P., Horita J. (2020). Bulk and position-specific isotope geochemistry of natural gases from the Late Cretaceous Eagle Ford Shale, south Texas. Mar. Pet. Geol..

[8] Zhao H., Luo H.Y., Zhang G.C., Wang Y., Wang X., Qin Y., Zhang D., Liu W. (2021). Study of the Mechanism for Identifying the Shale Gas ‘Sweet Spot’ Using the Reversed δ13C1-3 Series. Acta Geol. Sin..

[9] Pei L., Liu W., Guo Q., Wang X., Luo H., Wang Q. (2023). Genetic significance of carbon isotope curve types of methane, ethane, and propane in natural gas. Org. Geochem..

[10] Hunt A.G., Darrah T.H., Poreda R.J. (2012). Determining the source and genetic fingerprint of natural gases using noble gas geochemistry: A northern Appalachian Basin case study. AAPG Bull..

[11] Tilley B., McLellan S., Hiebert S., Quartero B., Veilleux B., Muehlenbachs K. (2011). Gas isotope reversals in fractured gas reservoirs of the western Canadian Foothills: Mature shale gases in disguise. AAPG Bull..

[12] Dai J., Zou C., Dong D., Ni Y., Wu W., Gong D., Wang Y., Huang S., Huang J., Fang C. (2016). Geochemical characteristics of marine and terrestrial shale gas in China. Mar. Pet. Geol..

[13] Shen B.-J., He Z.-L., Tao C., Shen J.-C., Hu Z.-Q., Li Z.-M., Cao Y.-H., Chen W. (2022). A mathematical diffusion model of carbon isotopic reversals inside ultra-tight Longmaxi shale matrixes. Pet. Sci..

[14] Liu C., Liu P., McGovern G.P., Horita J. (2019). Molecular and intramolecular isotope geochemistry of natural gases from the Woodford Shale, Arkoma Basin, Oklahoma. Geochim. Cosmochim. Acta.

[15] Wen Y., Zhang L., Li Y., Huntington K.W., Jin T., Schauer A.J., Wang C. (2022). Late Mesozoic elevation history of the north Taihang Mountains, China: Constraints from clumped isotope geochemistry. GSA Bull..

[16] Xia X., Tang Y. (2012). Isotope fractionation of methane during natural gas flow with coupled diffusion and adsorption/desorption. Geochim. Cosmochim. Acta.

[17] Wang X.F., Li X.F., Wang X.Z., Shi B., Luo X., Zhang L., Lei W., Jiang C., Meng Q. (2015). Carbon isotopic fractionation by desorption of shale gases. Mar. Pet. Geol..

[18] Stolper D.A., Lawson M., Davis C.L., Ferreira A.A., Neto E.V.S., Ellis G.S., Lewan M.D., Martini A.M., Tang Y., Schoell M. (2014). Formation temperatures of thermogenic and biogenic methane. Science.

[19] Gilbert A., Yamada K., Suda K., Ueno Y., Yoshida N. (2016). Measurement of position-specific 13C isotopic composition of propane at the nanomole level. Geochim. Cosmochim. Acta.

[20] Piasecki A., Sessions A., Lawson M., Ferreira A., Neto E.S., Eiler J.M. (2016). Analysis of the site-specific carbon isotope composition of propane by gas source isotope ratiomass spectrometer. Geochem. Cosmochim. Acta.

[21] Stahl W.J., Carey B.B. (1975). Source rock identification by isotope analyses of natural gases from fields in the Val Varde Delaware Basins, West Texas. Chem. Geol..

[22] Prinzhofer A., Hue A. (1995). Genetic and post-genetic molecular and isotopic fractionation in natural gas. Chem. Geol..

[23] Liu W.H., Xu Y.C. (1999). A two stage model of carbon isotopic fractionation in coal gas. Geochimica.

[24] Clayton C. (1991). Carbon isotope fractionation during natural gas generation from kerogen. Mar. Pet. Geol..

[25] Berner U., Faber E., Stahl W. (1992). Mathematical simulation of the carbon isotopic fractionation between huminitic coals and related methane. Chem. Geol. Isot. Geosci..

[26] Lorant F., Prinzhofer A., Behar F., Huc A.-Y. (1998). Carbon isotopic and molecular constraints on the formation and the expulsion of thermogenic hydrocarbon gases. Chem. Geol..

[27] Tang Y., Huang Y., Ellis G.S., Wang Y., Kralert P.G., Gillaizeau B., Ma Q., Hwang R. (2005). A kinetic model for thermally induced hydrogen and carbon isotope fractionation of individual n-alkanes in crude oil. Geochim. Cosmochim. Acta.

[28] Wang Y., Zhang S., Wang F., Wang Z., Zhao C., Wang H., Liu J., Lu J., Geng A., Liu D. (2006). Thermal cracking history by laboratory kinetic simulation of Paleozoic oil in eastern Tarim Basin, NW China, implications for the occurrence of residual oil reservoirs. Org. Geochem..

[29] Tian H., Xiao X., Wilkins R., Li X., Gan H. (2007). Gas sources of the YN2 gas pool in the Tarim Basin—Evidence from gas generation and methane carbon isotope fractionation kinetics of source rocks and crude oils. Mar. Pet. Geol..

[30] Pan C., Jiang L., Liu J., Zhang S., Zhu G. (2010). The effects of calcite and montmorillonite on oil cracking in confined pyrolysis experiments. Org. Geochem..

[31] Jia W., Wang Q., Liu J., Peng P., Li B., Lu J. (2014). The effect of oil expulsion or retention on further thermal degradation of kerogen at the high maturity stage: A pyrolysis study of type II kerogen from Pingliang shale, China. Org. Geochem..

[32] Xiang B., Li E., Gao X., Wang M., Wang Y., Xu H., Huang P., Yu S., Liu J., Zou Y. (2016). Petroleum generation kinetics for Permian lacustrine source rocks in the Junggar Basin, NW China. Org. Geochem..

[33] Shuai Y., Zou Y., Liu J. (2005). Carbon Isotope Modeling of Coal-derived Methane and Ethane from the Upper Paleozoic of the Ordos Basin, China. Geol. Rev..

[34] Shuai Y., Zou Y., Peng P. (2006). Kinetic modeling of stable carbon isotope ratios of ethane from coal in confined system and its significance in geological application. Geochimica.

[35] Liu J., Tang Y. (1998). Kinetics of early methane generation from Green River shale. Chin. Sci. Bull..

[36] Sweeney J.J., Burnham A.K. (1990). Evaluation of a Simple Model of Vitrinite Reflectance Based on Chemical Kinetics. AAPG Bull..

[37] Zhao W.Z., Wang Z.Y., Zhang S.C., Wang H., Zhao C., Hu G. (2005). Successive generation of natural gas from organic materials and its significance in future exploration. Pet. Explor. Dev..

[38] Li J., Ma W., Wang Y., Wang D., Xie Z., Li Z., Ma C. (2018). Modeling of the whole hydrocarbon-generating process of sapropelic source rock. Pet. Explor. Dev..

[39] Ni L.J., Zhang L.G., Ni J.F., Yuan W.K. (1995). Structural kinetic model of pyrolysis process of paraffins and its simulation. J. Chem. Ind. Eng..

[40] ZHANG H.M., ZHANG H.W., GU P.P., Zhao L. (2012). Molecular simulation of propane pyrolysis reaction. Acta Pet. Sin. (Pet. Process. Sect.).

[41] Liu W., Wang J., Tenger, Qin J., Zheng L. (2012). Stable carbon isotopes of gaseous alkanes as genetic indicators inferred from laboratory pyrolysis experiments of various marine hydrocarbon source materials from southern China. Sci. China Earth Sci..

[42] Tian H., Xiao X., Wilkins R.W., Tang Y. (2012). An experimental comparison of gas generation from three oil fractions: Implications for the chemical and stable carbon isotopic signatures of oil cracking gas. Org. Geochem..

[43] Li E., Pan C., Yu S., Jin X., Liu J. (2013). Hydrocarbon generation from coal, extracted coal and bitumen rich coal in confined pyrolysis experiments. Org. Geochem..

[44] Cao H.R. (2017). The Paleo-Environment of Source Rock Formation and Geological Evaluation of Shale Oil in the Songliao Basin.

[45] Wang Q., Zou H., Hao F., Zhu Y., Zhou X., Wang Y., Tian J., Liu J. (2014). Modeling hydrocarbon generation from the Paleogene source rocks in Liaodong Bay, Bohai Sea: A study on gas potential of oil-prone source rocks. Org. Geochem..

[46] Xiong Y.Q., Geng A.S., Liu J.Z. (2004). Kinetic modeling of carbon isotope fractionation of coal-derived methane. Geochimica.

[47] Xiong Y.Q., Geng A.S., Liu J.Z., Wang Y.P., Liu D.H., Jia R.F., Shen J.G. (2002). Kinetic simulating experiment combined with GC-IRMS analysis: Application to identification of effective source rock. Geochimica.

[48] Hill R.J., Tang Y., Kaplan I.R. (2003). Insights into oil cracking based on laboratory experiments. Org. Geochem..

[49] Gong S., Peng P.A., Lu Y.H., Xiao Z.X., Jia W.L., Wang Z.Q., Yu C.L., Liu D.H., Lu J.L., Liu J.Z. (2004). The second heating experiment of biodegraded asphalt sand. Chin. Sci. Bull..

[50] Yin Q., Song Z.G., Liu J.Z. (2010). Influences of sulfur on composition of oil cracked gas and carbon isotopes. Oil Gas Geol..

[51] Li J.K., Fang W., Zeng H.S., Liu W., Zou Y.R., Liu J.Z. (2011). Possible origins for inverse stable carbon isotopes of gaseous alkanes from the Xujiaweizi fault depression. Acta Pet. Sin..

[52] Tang Q.Y., Zhang M.J., Yu M., Zhang T.W., Liu J.Z., Zhang M.C. (2013). Pyrolysis constraints on the generation mechanism of shale gas. J. China Coal Soc..

[53] Tang Q.Y., Zhang M.J., Zhang T.W., Liu J.J., Yu M. (2014). Kinetic pyrolysis simulation of hydrocarbon generation in shale system: A case study on Pearl River Mouth Basin, China. Geochimica.

[54] Wang Q.T., Lu H., Gao L.H., Xiong P., Shen C.C., Liu J.Z., Peng P.A. (2013). Geochemical characterization of thermogenic gas during the simulation experiments of the mature Salgan Shale. J. China Coal Soc..

[55] Gao L., Schimmelmann A., Tang Y., Mastalerz M. (2014). Isotope rollover in shale gas observed in laboratory pyrolysis experiments: Insight to the role of water in thermogenesis of mature gas. Org. Geochem..

[56] Chen Y. (2015). Mechanisms and Evaluation of Shale Gas Generation from Organic-Rich Marine Shales.

[57] Gai H., Xiao X., Cheng P., Tian H., Fu J. (2014). Gas generation of shale organic matter with different contents of residual oil based on a pyrolysis experiment. Org. Geochem..

[58] Shao D., Ellis G.S., Li Y., Zhang T. (2018). Experimental investigation of the role of rock fabric in gas generation and expulsion during thermal maturation: Anhydrous closed-system pyrolysis of a bitumen-rich Eagle Ford Shale. Org. Geochem..

[59] Xia X.Y., Li C.Y., Zhao L. (1998). Influence of mixture on isotope indices in gas source discrimination. Pet. Explor. Dev..

[60] Qin Y., Zhang Y.S., Zhu Y.M., Fan B.H., Jiang B., Li T.Z. (2000). Lagging and reaction kinetic mechanism of hydrocarbonregeneration from organic matters in coals. Earth Sci. J. China Univ. Geosci..

[61] Jin Q., Wang X.H., Hu X.Q., Wang L., Wang J., Song G.Q. (2008). Kinetics of primary and secondary generation of coal-derived gases and its applications to genesis of natural gases found in Gubei area, Zhanhua Depression. Geochimica.

